# Cardiac mitochondrial metabolism during pregnancy and the postpartum period

**DOI:** 10.1152/ajpheart.00127.2024

**Published:** 2024-03-29

**Authors:** Emily B. Schulman-Geltzer, Kyle L. Fulghum, Richa A. Singhal, Bradford G. Hill, Helen E. Collins

**Affiliations:** Division of Environmental Medicine, Department of Medicine, Center for Cardiometabolic ScienceChristina Lee Brown Envirome Institute, University of Louisville, Louisville, Kentucky, United States

**Keywords:** female cardiac biology, ketone body, mitochondrial subunit complexes, physiological hypertrophy, solute transporters

## Abstract

The goal of the present study was to characterize changes in mitochondrial respiration in the maternal heart during pregnancy and after birth. Timed pregnancy studies were performed in 12-wk-old female FVB/NJ mice, and cardiac mitochondria were isolated from the following groups of mice: nonpregnant (NP), midpregnancy (MP), late pregnancy (LP), and 1-wk postbirth (PB). Similar to our previous studies, we observed increased heart size during all stages of pregnancy (e.g., MP and LP) and postbirth (e.g., PB) compared with NP mice. Differential cardiac gene and protein expression analyses revealed changes in several mitochondrial transcripts at LP and PB, including several mitochondrial complex subunits and members of the *Slc* family, important for mitochondrial substrate transport. Respirometry revealed that pyruvate- and glutamate-supported state 3 respiration was significantly higher in PB vs. LP mitochondria, with respiratory control ratio (RCR) values higher in PB mitochondria. In addition, we found that PB mitochondria respired more avidly when given 3-hydroxybutyrate (3-OHB) than mitochondria from NP, MP, and LP hearts, with no differences in RCR. These increases in respiration in PB hearts occurred independent of changes in mitochondrial yield but were associated with higher abundance of 3-hydroxybutyrate dehydrogenase 1. Collectively, these findings suggest that, after birth, maternal cardiac mitochondria have an increased capacity to use 3-OHB, pyruvate, and glutamate as energy sources; however, increases in mitochondrial efficiency in the postpartum heart appear limited to carbohydrate and amino acid metabolism.

**NEW & NOTEWORTHY** Few studies have detailed the physiological adaptations that occur in the maternal heart. We and others have shown that pregnancy-induced cardiac growth is associated with significant changes in cardiac metabolism. Here, we examined mitochondrial respiration and substrate preference in isolated mitochondria from the maternal heart. We show that following birth, cardiac mitochondria are “primed” to respire on carbohydrate, amino acid, and ketone bodies. However, heightened respiratory efficiency is observed only with carbohydrate and amino acid sources. These results suggest that significant changes in mitochondrial respiration occur in the maternal heart in the postpartum period.

## INTRODUCTION

The maternal heart adapts rapidly during pregnancy to meet the metabolic demands of the mother and her growing fetus. The increased workload and metabolic demand placed on the maternal heart results in cardiac growth. Few studies have interrogated the underlying metabolic mechanisms contributing to maternal cardiac growth. Changes in cardiac glucose metabolism during late pregnancy play a key role in pregnancy-associated cardiac growth (1–5). In addition, we showed that maternal cardiac growth, which was greatest during late pregnancy (LP) and postbirth (PB), was associated with significant increases in key glucose and ketone body metabolism enzymes, such as pyruvate dehydrogenase kinase 4 (PDK4) and 3-hydroxybutyrate dehydrogenase 1 (BDH1), along with increases in cardiac metabolites associated with anabolism (6). We also showed that hearts from both LP and PB dams had reduced expression of essential mitochondrial complex subunits, specifically those associated with complexes I and IV; however, we did not interrogate whether cardiac mitochondrial function was changed, nor did we examine myocardial mitochondrial substrate preference.

Although changes in mitochondrial metabolism play a critical role in cardiac growth in other physiological and pathological contexts (7–10), it is not clear how mitochondria remodel in the maternal heart during and after pregnancy. This is because changes in cardiac mitochondrial metabolism have yet to be interrogated in the maternal heart, leaving a significant gap in knowledge in the field. With the increased workload placed on the maternal heart, it is likely that changes in mitochondrial function occur to meet ATP demands. Understanding mitochondrial changes during pregnancy is important because mitochondrial dysfunction has been implicated in the development of pregnancy-associated cardiovascular diseases, such as preeclampsia (11–13); however, how maternal cardiac mitochondria respond to a normal, uncomplicated pregnancy is surprisingly unknown. Thus, to address this significant gap in knowledge, we examined how cardiac mitochondrial function and substrate preference change during pregnancy and the postpartum period.

## MATERIALS AND METHODS

### Experimental Animals

All procedures were approved by the Institutional Animal Care and Use Committee at the University of Louisville and followed the National Institutes of Health’s Guide for the Care and Use of Laboratory Animals. FVB/NJ female and male mice (10 wk old) were purchased from The Jackson Laboratory. The animals were placed on a 12-h:12-h light/dark cycle and received standard chow (Lab Diets No. 5010) and water ad libitum. Mice received 2 wk of acclimatization to the University of Louisville animal facilities before experimentation. All mice were paired for pregnancy studies at 12 wk of age. Mice were anesthetized with sodium pentobarbital (150 mg/kg ip) and euthanized by excision of the heart, consistent with the 2020 American Veterinary Medical Association Guidelines on Euthanasia. In addition, all experimental procedures adhered to the ARRIVE guidelines (14), and, where possible, investigators were blinded to the experimental group. All experimental samples were collected at the same time of day (i.e., 10:00 am; where lights go on at 6:00 am) to rule out time of day-dependent changes.

### Timed Pregnancy Protocol

Timed pregnancy studies were performed in primiparous mice, as previously described (6). The following groups were examined: nonpregnant, diestrus (NP); midpregnant (*day 8* of pregnancy, MP); late pregnant (*day 16* of pregnancy, LP); and 1-wk postbirth with lactation (PB). The PB mice were left with their respective litters and continued lactating and nursing their pups until 1-wk postbirth. Experimental measurements were made in mice following one pregnancy rather than multiple pregnancies in line with our previous studies (6).

### Tissue Harvest and Gravimetry

Tissue harvest and gravimetry were performed as described (6).

### Mitochondrial Isolation

Intact hearts (i.e., atria and ventricles) were excised and total heart weight was determined. Collected hearts were then washed of excess blood in ice-cold isolation buffer, containing (in mM) 220 mannitol, 70 sucrose, 5 MOPS, and 1 EGTA and 0.2% fatty acid-free BSA (pH 7.2), minced finely, and homogenized using a Potter-Elvehjem tube and Teflon pestle, using methods similar to those outlined in Fulghum et al. (15). Protein concentration was quantified using the Bio-Rad DC assay, and the crude yield was estimated by normalizing to total heart weight. Then, the isolated cardiac mitochondria were used for extracellular flux (XF) analysis. The remaining mitochondrial pellets were stored at −80°C for subsequent analysis of mitochondrial protein expression (as described in *Immunoblotting*).

### Extracellular Flux Analysis

Oxygen consumption rate (OCR) is the rate at which oxygen is consumed by a cell and is used to assess cellular metabolism and mitochondrial function. Therefore, to measure mitochondrial respiration, we recorded the OCR from isolated cardiac mitochondrial preparations using a Seahorse XFe96 analyzer (Agilent), essentially as described (15). Briefly, 2.5 µg of mitochondria in respiration buffer were loaded into individual wells of a Seahorse XFe96 plate. We assessed state 3 respiration (adenosine diphosphate (ADP)-stimulated respiration) using the following substrate combinations: glutamate-malate-ADP, succinate-rotenone-ADP, octanoyl carnitine-malate-ADP, pyruvate-malate-ADP, malate-ADP, and 3-hydroxybutyrate-malate-ADP. All substrates had a final concentration of 5 mM except for 3-OHB, which was 2.5 mM. Each substrate mix, except for succinate, contained malate (2.5 mM) and ADP (1 mM). Malate was added to ensure appropriate pyruvate dehydrogenase (PDH) activity and to prevent the loss of tricarboxylic acid (TCA) cycle intermediates (16). OCR measurements were obtained following baseline readings, and we included measurement cycles after injection of each substrate or metabolic modulator [e.g., oligomycin (1.5 µM), FCCP (1 µM), and rotenone (1.0 µM)/antimycin-A (10 µM)]. State 4 respiration was assessed by the OCR after oligomycin injection. OCR readings were subsequently normalized to protein content. Respiratory control ratio (RCR) is a measure of mitochondrial efficiency and was calculated as the ratio of state 3 to state 4 respiration.

### Immunoblotting

Stored mitochondrial pellets were solubilized in 100 µL of isolation buffer containing 1% Triton X-100, 2% SDS, and protease inhibitor (protease inhibitor cocktail; Sigma P8340) and then vortexed. Solubilized pellets were spun at 13,400 *g* for 20 min at 4°C. Lysates were prepared and immunoblotting was performed as described previously (6). With the exception of using 10 µg of protein per lane and precast gels (4–15% Criterion TGX Precast Midi Protein Gel, No. 5671085). We used primary antibodies specific for BDH1 (Atlas Laboratories, No. HPA030947, 1:20,000), PDK4 (Abcam, No. ab214938, 1:20,000), and voltage-dependent anion channel (VDAC; Cell Signaling No. 4661S, 1:20,000) and appropriate secondary antibodies (Cell Signaling, No. 7074S or No. 7076S at 1:10,000). Membranes were imaged using the Bio-Rad ChemiDoc imaging system. Protein expression was normalized to VDAC expression. Densitometric analyses were performed using BioRad Image laboratory software.

### Reexamination of Cardiac RNA-Seq Data Set

Our existing RNA-seq data set (6), obtained using polyA capture methods, was reexamined to focus on differentially expressed cardiac mitochondrial transcripts during pregnancy and the postpartum period. For reanalysis, we used the same bioinformatics methods per our published work with the exception of retaining ribosomal and mitochondrial genes that were previously filtered out. This new DESeq2 output was queried against a list of 1,182 *Mus musculus* nuclear and mitochondrial genes, curated from Mouse MitoCarta2.0 (1,158 genes) and the RNA sequencing data set (24 genes). A total of 1,141 nuclear and mitochondrial genes were extracted from DESeq2 normalized count output for all the experimental groups, and DESeq2 differential expression was examined for the following comparisons: MP vs. NP, LP vs. NP, PB vs. NP, LP vs. MP, and PB vs. LP. In addition, genes unique to only one comparison group were excluded, and a heat map and principal component analysis plot were generated for 128 shared mitochondrial genes based on normalized DESeq2 counts. DESeq2 guidelines were used to identify differentially expressed genes, and all *P* values were adjusted for testing multiple genes (Benjamini–Hochberg procedure; *q* ≤ 0.05). A *q* value of 0.05 was used to reduce the potential generation of false positive findings in our analyses. Functional enrichment analysis was performed using the clusterProfiler R package and MetaCore version 22.1 build 70800 to identify enriched Gene Ontology biological processes and KEGG pathways for each set of differentially expressed genes (DEGs; 17).

### Reexamination of Cardiac Proteomics

Our cardiac proteomics data set (6) was also reexamined against the Mouse MitoCarta2.0 (1158), similar to our transcriptomic data set (described earlier), which identified 718 mitochondrial proteins. These 718 mitochondrial proteins were subjected to statistical analyses with FDR set to 0.05. Because we previously observed minimal proteomic changes between NP and MP hearts (6), we examined the following comparisons: LP vs. NP and PB vs. NP.

### Statistical Analyses

**Figure 1.**
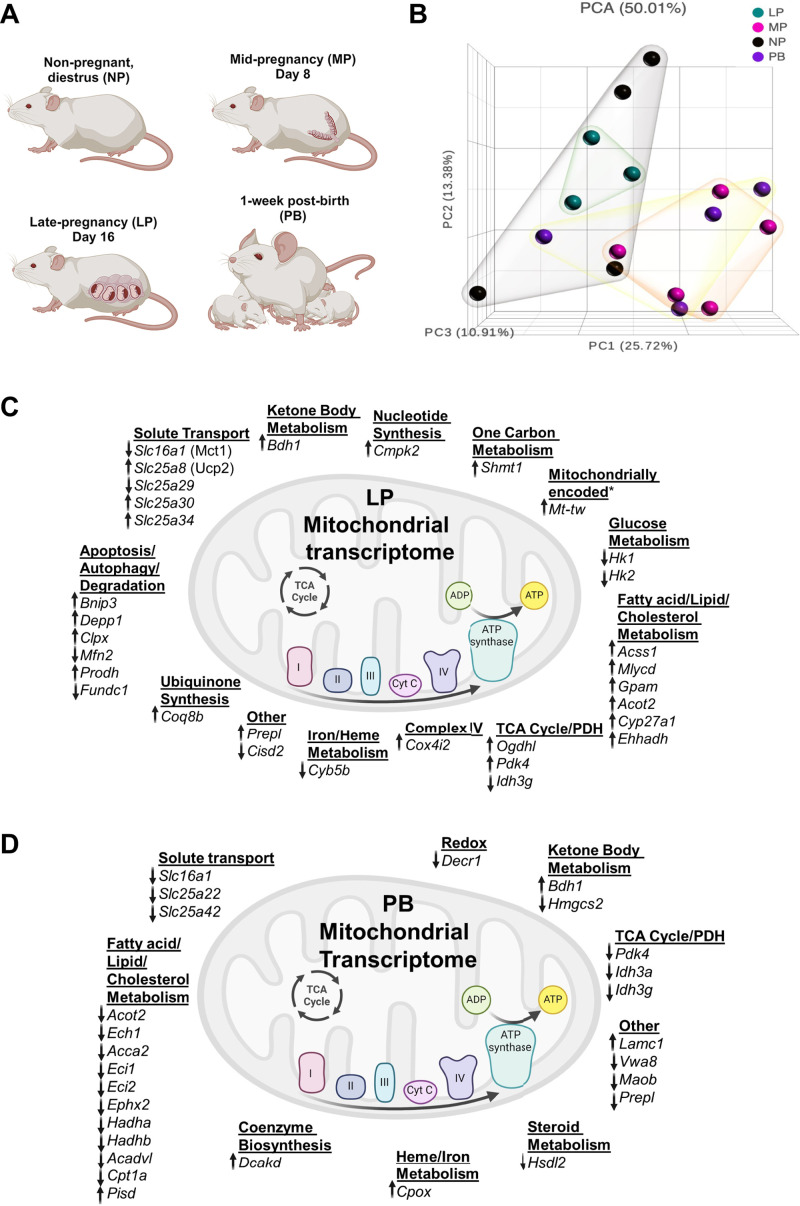
Examination of mitochondrial transcript expression in the pregnant and postpartum heart. We reexamined our preexisting RNA-seq data set (6) in which 1,158 *Mus musculus* nuclear and mitochondrial encoded genes were obtained from Mouse MitoCarta2.0 and queried within DEseq2 output. *A*: time points during pregnancy and postbirth examined. *B* and *C*: principal component analysis (*B*) and schematic of the upregulated and downregulated differentially expressed genes (DEGs; *C*) in the late pregnant (LP; *day 16*) hearts vs. nonpregnant hearts. *D*: schematic of upregulated and downregulated DEGs in hearts from mice 1-wk postbirth (PB; with lactation). *Mitochondrially encoded transcripts; *n* = 3–5 hearts/group. Figure was created using a licensed version of BioRender.com.

**Figure 2.**
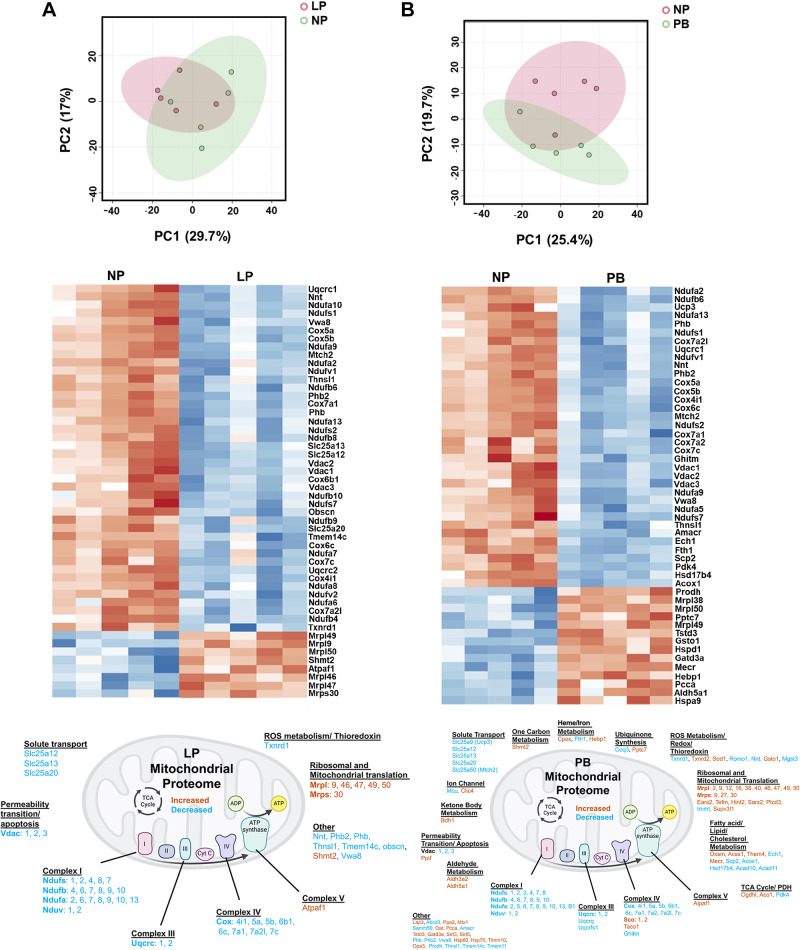
Examination of the mitochondrial proteome in the pregnant and postpartum heart. Our existing cardiac proteomics data set was reexamined to enable a targeted analysis of mitochondrial proteins against the Mouse MitoCarta2.0 database. *A*: principal component analysis (PCA) plot (*top*) and heat map showing top 50 mitochondrial proteins (*middle*), and schematic of major cardiac proteomic changes *(bottom*) between nonpregnant (NP) and late pregnant (LP) hearts. *B*: PCA plot (*top*), heat map showing the top 50 mitochondrial proteins (*middle*), and schematic of the major cardiac mitochondrial proteomic changes (*bottom*) between NP and postbirth (PB) hearts; *n* = 5 hearts/group. Figure was created using a licensed version of BioRender.com.

**Figure 3.**
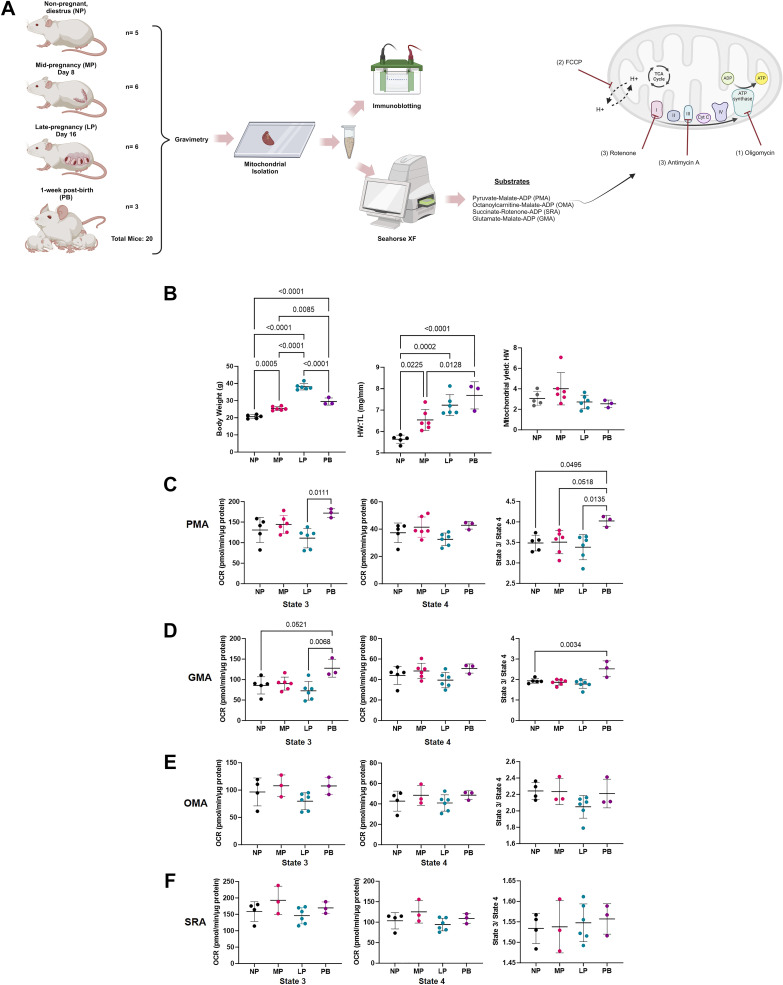
Increased pyruvate and glutamate respiration in isolated cardiac mitochondria from postpartum period hearts. *A*: main experimental time points for mouse-timed pregnancy study and downstream analyses in nonpregnant (NP), midpregnant (MP), late pregnant (LP), and 1-wk postbirth mice (PB; with lactation). *B*: body weight (*left*), heart weight:tibia length (HW:TL; *middle*), and normalized mitochondrial yield (*right*). Cardiac mitochondria were isolated from NP, MP, LP, and PB mice (with lactation) and subjected to extracellular flux analysis to determine state 3 and state 4 respiration (per *A*) in response to pyruvate-malate-ADP (PMA; *C*), glutamate-malate-ADP (GMA; *D*), octanylcarnitine-malate-ADP (OMA; *E*), and succinate-rotenone-ADP (SRA; *F*). *n* = 3–6 mice/group, one-way ANOVA followed by Tukey’s post hoc. All data presented are shown as means ± SD. Figure was created using a licensed version of BioRender.com.

**Figure 4.**
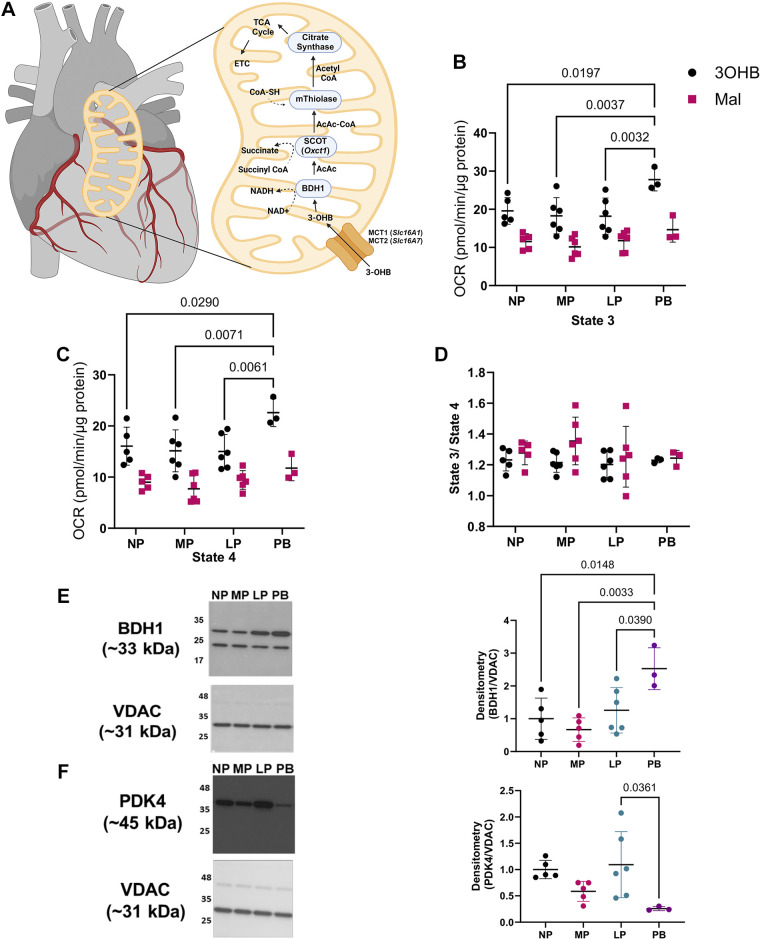
Isolated cardiac mitochondria from postpartum hearts respire more avidly to 3-hydroxybuytrate. *A*: 3-hydroxybutryrate metabolism. Cardiac mitochondria were isolated from nonpregnant (NP), midpregnant (MP), late pregnant (LP), and 1-wk postbirth mice (PB) and subjected to extracellular flux analysis to determine state 3 (*B*) and state 4 (*C*) respiration and the state 3:state 4 ratio (*D*) in response to 3-hydroxybutyrate-malate and malate. *E* and *F*: 3-hydroxybutyrate dehydrogenase 1 (BDH1; *E*) and pyruvate dehydrogenase kinase 4 (PDK4; *F*) protein expression in isolated mitochondria normalized to voltage-dependent anion channel (VDAC) expression. Representative, cropped immunoblots are shown. *n* = 3–6 hearts/group. Mixed effects analysis (Seahorse) and Immunoblot (one-way ANOVA followed by Tukey’s post hoc). All data presented are shown as means ± SD. Figure was created using a licensed version of BioRender.com.

Statistical significance was calculated using a one-way ANOVA followed by a Tukey’s post hoc test, where all groups were compared with each other. Where indicated, a mixed effects analysis was performed. All statistical analyses were performed using GraphPad Prism 10. *P* values of <0.05 were deemed statistically significant. All data are presented as means ± SD. All experimental data sets were completed with between 3 and 6 animals/group. The data presented in Figs. 1 and 2 are a reexamination of data previously published (6) and therefore reflect different cohorts of mice as those examined in Figs. 3 and 4.

## RESULTS

### Changes in Mitochondrial Gene Expression in the Maternal Heart

We had previously found that several mitochondrial complex subunit proteins are reduced in hearts of LP and PB mice (6); however, this specific analysis encompassed all transcriptomic changes, was aligned to an older version of the mouse genome, and excluded ribosomal and mitochondrial encoded genes. Therefore, to determine the full extent of changes in mitochondrial transcripts in the maternal heart, we reexamined our cardiac RNA-seq data set from FVB/NJ mice at NP, MP, LP, and PB (6) (Fig. 1*A*) to add in the mitochondrially encoded transcripts that were previously filtered out and aligned the data to the most recent mouse genome. Principal component analysis (Fig. 1*B*) showed distinct group separation between all four groups, highlighting distinct mitochondrial transcriptional profiles during these stages. Log2 fold changes of mitochondrial genes in LP vs. NP hearts, and PB vs. NP hearts are schematized in Fig. 1, *C* and *D*, respectively. For the purposes of the present study, we primarily focused on the LP and PB groups because in prior analyses, these groups had the greatest amounts of cardiac growth and also the changes in mitochondrial proteins were isolated to both LP and PB groups. Not surprisingly, many of the metabolic transcripts we identified previously were significantly increased in a similar manner (i.e., *Bdh1*, *Pdk4*, *Ogdhl*, etc.) (6). Reexamination revealed significant changes in many mitochondrial complex subunit-encoding transcripts and solute transporters responsible for bringing substrates into mitochondria, such as members of the *Slc* family (e.g., *Slc25a22*, *Slc25a29*, *Slc25a30*, *Slc25a34*, *Slc25a42*, and *Slc16a1*). In addition, there were decreases in *Slc16a1* (as in Fig. 1, *C* and *D*), which encodes MCT1, responsible for transporting lactate and ketone bodies. Not surprisingly, pathway analyses identified several key metabolic pathways, including ketone body metabolism, as changed in the maternal heart (data not shown).

### Mitochondrial Protein Expression in the Maternal Heart

To determine whether the identified changes in mitochondrial transcripts were reflected by similar changes in protein expression, we next reanalyzed our previously published cardiac proteomic data set from FVB/NJ mice at NP, MP, LP, and PB to focus on mitochondrial proteins, similar to the reexamination performed for transcriptomics. Realignment of the proteomic data identified 718 mitochondrial proteins across all four groups, and these proteins were further analyzed. We specifically focused on the LP vs. NP and PB vs. NP comparisons, as we had previously observed a reduction in mitochondrial complex subunit proteins in these comparisons. In addition, at the FDR cutoff of 0.05, the comparison of MP vs. NP did not yield any significant changes. Reexamination did reveal significant changes in mitochondrial protein expression between LP vs. NP (i.e., 53 proteins) and PB vs. NP (i.e., 124 proteins). For the comparisons of LP vs. NP (Fig. 2*A*) and PB vs. NP (Fig. 2*B*), we observed significant changes in several Slc25 transporters once again. However, when compared with the transcriptomics analysis, a considerably larger number of mitochondrial complex subunit proteins were found to be downregulated in both comparisons, consistent with our previous observations (6).

### Pregnancy-Associated Physiological Changes

To determine the extent to which cardiac mitochondrial function is altered during pregnancy and the postpartum period, we performed timed pregnancy studies in FVB/NJ mice at the following experimental time points: nonpregnant, diestrus (NP), midpregnant (*day 8* of pregnancy, MP), late pregnant (*day 16* of pregnancy, LP), and 1-wk postbirth with lactation (PB) (Fig. 3*A*). Consistent with our previous findings, we observed an increase in body weight throughout each stage of pregnancy (e.g., MP and LP) and postbirth (PB), which peaked at LP (Fig. 3*B*, *left*). Also consistent with our previous findings (6), we found that normalized heart weight (i.e., heart weight:tibia length; HW:TL) increased throughout all stages of pregnancy and postbirth, peaking at PB (Fig. 3*B*, *middle*). This peak in HW:TL at PB, although obtained from a smaller *n* of mice, was consistent with our previous findings in a larger cohort of mice (6). Not surprisingly, we also observed increased average pup weight at each successive stage of pregnancy and postbirth, consistent with appropriate fetal and newborn growth (data not shown). Because the increase in maternal heart weight could be a consequence of increased number of pups in utero or increased lactation burden as a result of increased litter sizes, we recorded number of pups per litter. Despite an increase in cardiac size with each pregnancy and postpartum time point (e.g., MP to LP to PB), there were no significant changes in litter size between MP and LP (10.67 ± 0.82 vs. 9.83 ± 1.47, *P* = 0.47) and LP and PB (9.83 ± 1.47 vs. 7.67 ± 1.15, *P* = 0.06). There was, however, a significant decrease in litter size between MP and PB groups (*P* < 0.01), which could be the result of in utero reabsorption or could be the result of pup death shortly after birth. However, we did not observe any evidence to suggest the latter. Together, these data recapitulate our previous finding that maternal heart size increases during pregnancy and postpartum, with more significant cardiac growth occurring during LP and PB.

### Changes in Maternal Cardiac Mitochondrial Respiration during Pregnancy and Postpartum

We next determined whether pregnancy-induced cardiac growth is associated with alterations in cardiac mitochondrial function. In support of this notion, we have previously observed the downregulation of several key mitochondrial complex subunits in the hearts of LP and PB mice (6), which are time points associated with pregnancy-associated cardiac growth; however, these studies did not interrogate the consequence of such changes on mitochondrial respiration. Therefore, we first isolated cardiac mitochondria from NP, MP, LP, and PB mice (Fig. 3*A*) and determined crude mitochondrial yield. We observed no significant differences in mitochondrial yield between groups (Fig. 3*B*, *right*). To examine state 3 mitochondrial respiration, we performed extracellular flux (XF) analysis following the provision of pyruvate-malate-ADP (PMA), glutamate-malate-ADP (GMA), octanylcarnitine-malate-ADP (OMA), and succinate-rotenone-ADP (SRA), followed by provision of oligomycin to ascertain state 4 respiration. In response to PMA, state 3 respiration was significantly increased in mitochondria from PB hearts when compared with LP hearts (Fig. 3*C*). The respiratory control ratio (RCR), an indicator of mitochondrial coupling, was calculated through the ratio of state 3 to state 4 respiration. In response to PMA, mitochondria from PB hearts had a significantly higher RCR vs. LP mitochondria, and a similar increase was observed between PB and NP/MP mitochondria. Like with PMA, PB mitochondria had significantly increased state 3 respiration vs. LP mitochondria in response to GMA (Fig. 3*D*). No significant changes were observed in state 4 respiration in response to GMA in any group; however, we observed significantly higher RCR in PB mitochondria respiring on GMA compared with all other groups (i.e., NP, MP, and LP). We observed no appreciable changes in state 3 respiration, state 4 respiration, or RCR in mitochondria from all groups in response to both OMA (Fig. 3*E*) and SRA (Fig. 3*F*). Together, these data suggest that PB mitochondria respire more avidly with pyruvate (i.e., PMA) and glutamate (i.e., GMA) and are more well coupled than mitochondria from NP, MP, or LP mice.

### Maternal Cardiac Respiration in Response to Ketone Body Provision

We have previously shown that cardiac 3-hydroxybutyrate dehydrogenase 1 (BDH1) expression increases in the maternal heart throughout pregnancy and postpartum (6). In the same study, we showed that cardiac levels of 3-hydroxybutyrate (3-OHB) were significantly elevated during pregnancy (6). In addition, circulating 3-OHB levels increase during late pregnancy in many mammalian species (18–21). Liver-derived, circulating 3-OHB can enter cardiac mitochondria through MCT1 (i.e., *Slc16A1*), MCT2 (i.e., *Slc16A2*), or via free diffusion (22), where it is converted by BDH1 to acetoacetate (AcAc), culminating in the generation of acetyl CoA for use in the TCA cycle (Fig. 4*A*). Given the increase in BDH1 expression in the maternal heart, we next determined respiration of mitochondria isolated from the maternal heart following provision of 2.5 mM 3-OHB. We found that there was a significant increase in state 3 respiration in PB mitochondria in response to 3-OHB when compared with NP, MP, or LP mitochondria, which we did not observe in any group in the presence of malate alone (Fig. 4*B*). A similar trend was observed with state 4 respiration, whereby we observed an increase in state 4 respiration in PB mitochondria vs. NP, MP, or LP mitochondria in response to 3-OHB, which was not observed in any group in the presence of malate alone (Fig. 4*C*). There were no significant changes in RCR between the groups (Fig. 4*D*). This suggests that although cardiac PB mitochondria respire more avidly on 3-OHB, there is no change in mitochondrial efficiency compared with cardiac mitochondria from nonpregnant mice or pregnancy phases. Given the increased mitochondrial respiration with 3-OHB in PB mitochondria, we next examined the expression of BDH1 using the remaining mitochondrial protein from respiration experiments. Although our previous studies show that, in whole cardiac homogenates, cardiac BDH1 protein expression is higher in MP, LP, and PB hearts (6), we find in isolated mitochondria that the abundance of BDH1 is significantly increased in PB mitochondria when compared with all other groups (Fig. 4*E*). In addition, we find lower PDK4 abundance in PB mitochondria compared with LP mitochondria (Fig. 4*F*). Overall, these findings are supportive of the increased respiration we observe in PB mitochondria in response to both 3-OHB and pyruvate.

## DISCUSSION

The maternal heart undergoes remarkable structural and metabolic adaptations during and after pregnancy to accommodate the profound physiological changes associated with gestation. These adaptations are crucial for meeting the increased demands on the cardiovascular system imposed by the growing fetus and the altered maternal metabolic state. Structurally, the heart undergoes hypertrophy, changes in chamber dimensions, and alterations in vascular function to enhance cardiac output. Metabolically, there is a shift in substrate utilization that appears to change depending on the stage of pregnancy (1, 9, 23). Nevertheless, several significant gaps persist in our knowledge; remaining unclear are the metabolic mechanisms that orchestrate structural and functional cardiac remodeling during pregnancy and their implications for maternal cardiovascular health. Specifically, there are currently no studies that examine cardiac mitochondrial function during pregnancy and the postpartum period. In this study, we used a murine model of pregnancy to characterize changes in metabolic transcripts and proteins and to understand the extent to which the state of pregnancy alters respiration in isolated mitochondria.

The metabolic changes that occur during pregnancy integrate several factors, including changes in substrate availability, the expression of substrate transporters and metabolic enzymes, and circulating hormones. Pregnancy studies in both animal models and humans demonstrate higher circulating levels of fatty acids, triglycerides, lactate, and ketone bodies (1, 24), which would be thought to increase their uptake and oxidation by the heart, although this has not been studied in appreciable detail. Furthermore, the maternal heart appears to demonstrate augmented fatty acid utilization rates and lower rates of glucose oxidation (1, 2, 24), which has been shown to contribute to remodeling of the heart during pregnancy. Despite our previous findings showing remarkable changes in the cardiac metabolome, transcriptome, and proteome during pregnancy (6), it remains unclear how mitochondrial substrate preference and activity are altered over the course of pregnancy. Moreover, our previous analyses of transcriptomics data used common practices for data analysis, which typically omit mitochondrially encoded genes, thereby limiting understanding of how mitochondria change over the course of pregnancy.

Targeted reexamination of our existing transcriptomic and proteomic data sets showed that relatively few mitochondrial proteins or substrate transporters change at midpregnancy, but that striking differences in substrate and metabolite transporters, mitochondrial metabolic enzymes, and electron transport chain components occur in late pregnancy and in the postpartum period. In particular, late pregnancy was associated with increases in key fatty acid metabolism transcripts (e.g., *Acss1*, *Mlycd*, *Gpam*, *Acot2*, *Cyp27a1*, and *Ehhadh*), ketone body metabolism transcripts (e.g., *Bdh1*), alongside changes in solute transporters (e.g., *Slc16a1*, *Slc25a8*, *Slc25a29*, *Slc25a30*, and *Slc25a34*), and pyruvate metabolism regulators or machinery (e.g., *Pdk4* and *Ogdhl*). In the postpartum period, we found that key fatty acid metabolism transcripts were significantly decreased (e.g., *Hadha*, *Hadhb*, *Cpt1a*, *Acadvl*, *Ech1*, *Acot2*, *Acca2*, *Eci1*, and *Eci2*), alongside reductions in solute transporters (e.g., *Slc16a1*, *Slc25a22*, and *Slc25a42*) and regulators of mitochondrial pyruvate metabolism (e.g., *Pdk4*). Conversely, we find that the ketone body metabolism transcript, *Bdh1*, was increased throughout pregnancy and postbirth. In addition, in line with our previous findings, reexamination of the cardiac proteome at late pregnancy and postbirth revealed significant decreases in mitochondrial complex subunits (e.g., subunits of complexes I, III, and IV) and increases in key proteins involved in ribosomal and mitochondrial translation (e.g., Mrpl and Mrps proteins). These findings suggest remarkable alterations in metabolic components related to mitochondrial function; however, these analyses were from whole heart samples, and it remains unclear how mitochondria, at the isolated organellar level, change during pregnancy.

Given that few studies have addressed how mitochondria remodel during pregnancy, and that there have been no studies that address respiratory changes in mitochondria isolated from the maternal heart, we next examined respiration in isolated mitochondria at different stages of pregnancy. As in our previous study (6), we found that maternal heart size increased during all stages of pregnancy and postbirth. Mitochondria were then isolated from these hearts, and mitochondrial respiration was determined in response to fatty acid, carbohydrate, amino acid, and ketone body sources. Surprisingly, we found that mitochondria isolated from pregnant or postpartum mice do not have a higher intrinsic capacity to respire on octanoylcarnitine, indicating that the higher levels of cardiac fatty acid oxidation that occur in vivo during pregnancy (1, 4) are independent of changes in mitochondrial substrate preference and are likely governed by increased circulating fatty acid levels or changes in sarcolemmal fatty acid transport. Given that numerous studies have shown a decrease in cardiac glucose oxidation in the latter stages of pregnancy (2–5), we expected to find a diminished capacity of mitochondria from LP hearts to respire on pyruvate. Although we noticed a trend toward decreased respiration on pyruvate in LP, we found a clear increase in the ability of PB hearts to respire on pyruvate, which is supported by diminished levels of PDK4 in isolated mitochondria from postpartum hearts (Fig. 4*F*). Similarly, we observed in PB mitochondria higher levels of glutamate-supported respiration, which could be due to elevated levels of glutamate dehydrogenase or glutamate transporters. However, this is unlikely to be the result of changes in Aralar (e.g., *Slc25a12*) protein expression because this was decreased in both late-pregnant and postbirth hearts. Because cardiac mitochondrial content affects metabolic capacity of the myocardium, it is possible that higher levels of cardiomyocyte mitochondria could impact maternal heart bioenergetics; however, our assessment of crude yield showed no differences between groups, suggesting that mitochondrial number is unlikely to be different. Moreover, we found no significant differences in mitochondrial proteins, such as VDAC, in whole heart homogenates from pregnant or postbirth dams, which appears to indicate that mitochondrial abundance does not change during pregnancy. These findings indicate that, in isolated mitochondria, the proclivity of mitochondria from postpartum hearts to respire on pyruvate and glutamate is increased, with little change in respiration during mid- or late pregnancy.

Although ketone bodies increase in circulation during pregnancy (18–21, 25), the extent to which the maternal heart uses ketone bodies as an energy source has not been examined in detail. Ketone body oxidation contributes ∼5–15% of ATP production in healthy, nonpregnant mammals (24). We found that isolated mitochondria from postpartum hearts respire more avidly on 3-OHB than mitochondria isolated at other stages of pregnancy. However, this respiration was less efficient than that of carbohydrate (pyruvate) and amino acid (glutamate) sources, as indicated by lower RCR values (a metric of oxidative phosphorylation efficiency). Analogous findings have been documented in the context of pathological cardiac hypertrophy, whereby there is increased availability of ketone bodies and increased oxidation of 3-OHB, but metabolic efficiency is not improved (26, 27). Although there is no increase in efficiency with 3-OHB-supported oxidative phosphorylation, this does not mean that it fails to impact metabolism. Our data support the interpretation that the maternal heart shows an increased capacity to oxidize 3-OHB during the postbirth phase, which would likely support respiration and spare other substrates (e.g., glucose, fatty acids) for other purposes (e.g., biosynthetic pathways, nutritional needs of pups, etc.). In support of this, a reduction in cardiac glucose oxidation has been shown to be necessary for pregnancy-associated cardiac growth (2), which could mean that in the maternal heart glucose may be directed to support biosynthetic pathways.

Surprisingly, we observed that the transcript encoding MCT1 (i.e., *Slc16a1*), responsible for transporting lactate and ketone bodies, was decreased in the maternal heart at LP and PB. Of note, MCT1 deficiency is associated with ketoacidosis (28, 29), and increases in cardiac MCT1 are associated with heart failure (30), suggesting that fluctuations in the ability of ketones to enter the mitochondria for oxidation could result in pathology. Nevertheless, ketone bodies have been shown to enter the mitochondria via free diffusion (22) so it remains unclear whether the changes in MCT1 affect ketone body metabolism in cardiomyocytes. Although the first enzyme in 3-hydroxybutyrate metabolism, i.e., BDH1, is upregulated in the heart during late pregnancy (6), we found that isolated mitochondria from LP hearts do not show elevated BDH1; rather, mitochondria from PB hearts demonstrated elevated BDH1, which could in part underlie their increased proclivity to respire on 3-hydroxybutyrate. Regardless, further work is required to determine the relevance of changes in ketone body metabolism in the maternal heart in the context of cardiac growth and metabolism.

### Limitations

In the present study, some of the examined observations stem from a smaller sample size, i.e., PB group, which could be considered a limitation. However, we found that, even with an *n* = 3, maternal cardiac mitochondria derived at PB showed convincingly higher levels of ketone body-supported respiration compared with the other groups (*P* < 0.005), which coincided with higher BDH1 abundance. Thus, these complementary data indicate that PB mitochondria have a higher capacity to oxidize 3-hydroxybutyrate.

In addition, we used a medium-chain fatty acylcarnitine (i.e., octanoylcarnitine) to assess respiration on fat sources, so it is unclear whether maternal cardiac mitochondria respire differently when provided longer chain acylcarnitines. It has been shown previously that the long-chain acylcarnitine, palmitoylcarnitine, can significantly reduce glucose and lactate oxidation in isolated male rat mitochondria (31); with the lack of studies on maternal cardiac metabolism, it is unclear whether this would yield comparable results. However, given that we have previously observed increases in cardiac acylcarnitines in the maternal heart (6), this increase in the availability acylcarnitines could contribute to the metabolic changes we observe in terms of substrate preference in the maternal heart. Similarly, we did not measure the extent to which mitochondria respire on acetoacetate (AcAc), the second most abundant ketone body in circulation. We chose not to examine AcAc in the context of these studies because preliminary dose-response studies of AcAc in isolated mitochondria did not yield any appreciable respiration at physiologically relevant concentrations (data not shown).

We used primiparous mice in the present studies to examine the effect of a single pregnancy-lactation cycle on cardiac mitochondrial function. However, our studies did not directly examine whether the mitochondrial remodeling we observe reverses following lactation to nonpregnant levels, nor did we examine the impact of multiple pregnancy-lactation cycles on mitochondrial remodeling and function. Our studies indicate that, despite being nonpregnant groups, both NP and PB groups differ substantially with respect to virtually all examined parameters, (e.g., heart size, mitochondrial function) with only pregnancy and lactation differing between both groups. Studies have shown that following birth the heart remodels to a prepregnancy-like size, following the completion of lactation and the reversion of pregnancy-associated hemodynamic changes (32–37); however not all studies have shown this (38). Furthermore, in none of these studies were cardiac metabolism or mitochondrial metabolism examined, so the consequence of pregnancy on long-term mitochondrial remodeling and function remains unclear and is the focus of ongoing studies.

Another limitation is that we did not assess how lactation influences mitochondrial activity after pregnancy. Postpartum mice in the present studies were maintained with their respective litters and were actively lactating. Because lactation is a highly ketogenic state (18, 39), the increase in cardiac mitochondrial respiration and increased BDH1 expression in PB hearts may result from increased ATP demand or substrate availability (e.g., 3-OHB). Indeed, recent studies suggest that the high energy demand imposed by lactation stimulates high-energy remodeling of mitochondrial function to help facilitate milk production while increasing ATP levels (40); however, those studies were limited to the examination of liver and skeletal muscle mitochondria (40). Ideally, future studies will examine the contributions of lactation to mitochondrial function using mice in which litters are removed after birth to simulate a reduction in lactation or using mice in which key lactogenic signaling components are disrupted. In addition, it is possible that the sex of the pups could influence maternal metabolic physiology; however, due to mixed-sex litters, it could be difficult to tease apart the influence of fetal biological sex on maternal cardiovascular health.

Finally, the present studies did not address whether mitochondrial morphology is altered during or after pregnancy. Notably, we found several transcripts associated with mitochondrial dynamics that were altered in the maternal heart, such as *Mfn2* and *Bnip1*. Given that mitochondrial dynamics have been shown to regulate fat oxidation capacity (41), future studies that address how mitochondrial ultrastructure (e.g., fission and fusion) regulates metabolic and structural cardiac remodeling during and after pregnancy could yield additional insights.

### Conclusions

Mitochondria from the postpartum murine heart respire avidly in the presence of carbohydrate, amino acid, and ketone bodies; however, heightened efficiency is observed only with carbohydrate (pyruvate) and amino acid (glutamate) sources. Although these results are descriptive, the findings of the study provide insights on maternal cardiovascular adaptations and changes in mitochondrial function during and after pregnancy. These studies indicate that the maternal heart can use ketone bodies as a fuel source, which could be an important way of maintaining mitochondrial metabolism during increased cardiac demand, such as during pregnancy and lactation. Whether the differences in mitochondrial respiration in postpartum hearts are due to lactation, differences in the hormonal milieu, or remodeling of specific mitochondrial complex subunits requires additional study. In addition, determining the importance of mitochondrial changes in the postpartum heart will likely yield significant insights into potential mechanisms that contribute to pregnancy-associated cardiovascular diseases, such as preeclampsia and peripartum cardiomyopathy. Understanding long-term maternal mitochondrial adaptations could contribute to not only the protective benefit of a healthy pregnancy on long-term female cardiovascular health (42) but could also in part contribute to the long-term disease risk in women who have had a pregnancy-associated cardiovascular complication (43). Understanding how these changes in mitochondrial respiration in PB hearts are impacted in the setting of reduced cardiac oxidation of ketone bodies (i.e., with genetic deletion of ketone body catabolic enzymes, such as *Bdh1* or *Oxct1*) will likely yield mechanistic insights into the importance of ketone bodies in the remodeling and health of the maternal heart.

## DATA AVAILABILITY

Data will be made available upon reasonable request.

## GRANTS

This work was funded by National Institutes of Health Grants HL163003 (to H.E.C.), HL147844 (to B.G.H.), HL163818 (to H.E.C.), and GM127607 (to H.E.C.); American Heart Association Grant 23TPA1141824 (to B.G.H.); the Jewish Heritage Fund For Excellence (JHFE) Faculty Support Grant (to H.E.C.); and the JFHE Research Enhancement Award (to H.E.C.).

## DISCLOSURES

Other than the listed Research Grant Funding, the authors have no additional conflicts of interest, financial or otherwise, to disclose.

## AUTHOR CONTRIBUTIONS

B.G.H. and H.E.C. conceived and designed research; E.B.S.-G., K.L.F., and H.E.C. performed experiments; E.B.S.-G., K.L.F., R.A.S., and H.E.C. analyzed data; E.B.S.-G., R.A.S., B.G.H., and H.E.C. interpreted results of experiments; E.B.S.-G., R.A.S., and H.E.C. prepared figures; H.E.C. drafted manuscript; E.B.S.-G., K.L.F., R.A.S., B.G.H., and H.E.C. edited and revised manuscript; E.B.S.-G., K.L.F., R.A.S., B.G.H., and H.E.C. approved final version of manuscript.
